# 

**Published:** 1888-09

**Authors:** 


### Society Meetings.

THE MISSISSIPPI VALLEY MEDICAL ASSOCIATION meets at St. Louis, September 11, 12 and 13, 1888. The programme includes many papers and discussions of importance. The first day will be given to the discussion of abdominal surgery; the second day to infant-feeding and some obstetric subject; the third day will be taken up with volunteer papers and some neurological subject. The Society cordially invites all members of the profession to be present.

J. LUCIUS GRAY, *Secretary.*

---

THE CONGRESS OF AMERICAN PHYSICIANS AND SURGEONS will hold its first annual meeting at Washington, September 18, 19 and 20, 1888. A dinner will be given to the guests of the Congress at Willard's hotel, at 7.30 P. M., September 17th.

---

THE SOUTHERN SURGICAL AND GYNECOLOGICAL ASSOCIATION will hold its first annual meeting at Birmingham, Ala., September 11, 12 and 13, 1888.

---

THE AMERICAN PHARMACEUTICAL ASSOCIATION will hold its thirty-sixth annual meeting in Detroit, commencing September 3, 1888.

---

THE AMERICAN RHINOLOGICAL ASSOCIATION will hold its sixth annual meeting in Cincinnati, September 12, 13 and 14, 1888.

### BOOKS AND PAMPHLETS RECEIVED.

Tennessee State Board of Health. Bulletin No. 10, Vol. III.

Memoir of Dr. A. F. Erich. By Dr. George H. Rohé, Baltimore.

New York State Board of Health. Monthly Bulletin. July, 1888.

Board of Health of Newport, R. I. Annual Reports for 1885–6–7.

Same. Reports of deaths and contagious diseases for July, 1888.

Pennsylvania State Board of Health. Circular on Camp Hygiene. 1888.

Yale University. Report of the Observatory Managers, 1886–7–8.

Transactions of the Medical Society of the State of West Virginia. 1888.

Cornell University. Bulletins I. and II. of the Agricultural Experiment Station.

Antipyrine. By Dr. Benj. Marshall. Author's reprint. San Francisco. 1888.

Chart. An Epitome of the Cranial Nerves. By Dr. Wheelock Rider, Rochester.

Exopthalmic Goitre. By Dr. Augustus A. Eshner. Prize Essay, Jefferson Medical College. 1888.

Conditions Rendering Diagnosis Difficult in Pelvic and Abdominal Diseases. Dr. T. B. Harvey, Indianapolis.

The Neural and Psycho-Neural Factor in Gynecic Disease. By Dr. C. H. Hughes. Author's reprint, St. Louis. 1888.

Footprints of a Profession; or, Ethics in Materials and Morals. By Horatio C. Meriam, D. M. D., St. Louis. 1888.

Michigan State Board of Health. Proceedings and Addresses at a Sanitary Convention held at Owosso, November 22 and 23, 1887.

Same. Health in Michigan. Bulletin for July, 1888.

Weekly Abstracts of Sanitary Reports. By the Surgeon-General U. S. Marine Hospital Service. Abstracts 31, 32, 33, 34. August, 1888.

Report of Senate Committee on Post-Offices and Post-Roads on Senate Bill 3329, regarding rates of postage on third and fourth class mail matter.

University of Nebraska. First Report from the Patho-Biological Laboratory. Southern Cattle Plague, and Yellow Fever. By Dr. Frank S. Billings, Director.

Rectal Insufflation of Hydrogen Gas. An infallible test in the diagnosis of visceral injury of the gastro-intestinal canal in penetrating wounds of the abdomen. By Dr. N. Senn, Milwaukee. Author's reprint. 1888.

The History of Abdominal Section in Albany, with a report of seventy-five cases. By Dr. A. Vander Veer, Albany. Author's reprint. 1888.

Intestinal Obstruction. Same author.

L'Immunité par les Vaccins Chimiques. Prévention de la Rage par le Vaccin Tanacétique ou le Chloral, par le Dr. H. Peyraud (de Libourne), Paris. 1888.

---

### COLLEGE ANNOUNCEMENTS RECEIVED. 1888-9

College of Pharmacy, Buffalo

Gross Medical College, Denver.

Missouri Dental College, St. Louis.

Eclectic Medical College, New York.

Texas Medical College and Hospital.

Medical Department, Niagara University.

The Medico-Chirurgical College, Philadelphia.

The Medical Department, University of Buffalo.

New York Post-Graduate Medical School and Hospital.

Meharry Medical Department, Central Tennessee College, Nashville.

